# State Firework Legislation and Pediatric Hand Trauma

**DOI:** 10.1001/jamanetworkopen.2025.54594

**Published:** 2026-01-16

**Authors:** Michael F. Catanzaro, Sandra V. Kotsis, Wenchu Pan, Lu Wang, Kevin C. Chung

**Affiliations:** 1 Section of Plastic Surgery, Department of Surgery, Michigan Medicine, Ann Arbor; 2 Department of Biostatistics, School of Public Health, University of Michigan, Ann Arbor

## Abstract

This cohort study investigates the association of state legislation and patient factors with odds of firework-related hand trauma among pediatric patients.

## Introduction

Hand and upper-extremity injuries are the most common presentation of firework injuries,^1^ incurring substantial long-term morbidity.^2^ Over the last decade, several states lifted restrictions on fireworks available for consumer purchase. Subsequently, firework-related injuries increased, particularly in the pediatric population.^2,3^ This study characterizes the association of state legislation and patient factors with pediatric firework-related hand trauma.

## Methods

This cohort study was considered not regulated by the University of Michigan Institutional Review Board and exempt from consent because the study was not regulated as human participant research per 45 CFR §46.102 and follows the STROBE reporting guideline. Data were obtained from the Pediatric Health Information System, a database from participating US pediatric hospitals.^4^ Data from 49 hospitals in 26 states were included.

We included patients aged 18 years and younger from 2015 to 2024 seen as inpatients or in the emergency department. Patients were selected using an *ICD-9* or *ICD-10* code specifying injuries caused by fireworks and a hand injury code (eTable in Supplement 1). A comparison group had similar hand injuries not owing to fireworks.

We collected state data regarding legal firework purchase age.^5^ Two study team members (M.F.C. and S.V.K.) independently categorized each state as restrictive or unrestrictive regarding permitted and prohibited fireworks.^6^ Results were compared and discrepancies resolved. We verified whether the law changed during the study period.

Our multilevel logistic regression model set the dependent variable as whether the hand injury was owing to fireworks. We checked for variable collinearity and performed a sensitivity analysis. We included age, gender, patient-defined race and ethnicity, living in an urban area, family income, injury occurring in July, state legal firework purchase age, state firework restrictions, and a random intercept to account for within-state correlation. Analysis was conducted in R version 4.2.3 (R Project for Statistical Computing) with a 2-sided *P* < .05 considered significant.

## Results

Among 37 573 patients (41.4% female; mean [SD] age, 4.7 [5.0] years; 23.0% African American, 55.1% White, and 17.5% other; 20.6% Hispanic) there were 645 firework hand injury cases and 36 928 nonfirework hand injury cases (Figure). For every 1-year increase in age, the likelihood of a firework hand injury significantly increased (odds ratio [OR], 1.09; 95% CI, 1.07-1.11; *P* < .001) (Table). Females had decreased odds (OR, 0.51; 95% CI, 0.42-0.61; *P* < .001) of firework injury, controlling for other covariates. African American patients had increased odds (OR, 1.37; 95% CI, 1.12-1.69; *P* = .003) of firework injury compared with White patients. Patients with family income below the state median had increased odds (OR, 1.28; 95% CI, 1.07-1.52; *P* = .006). Living in an urban area was not associated with firework injury. States with a legal purchase age of 17 years or older had decreased odds of firework injury (OR, 0.45; 95% CI, 0.22-0.94; *P* = .03) compared with 15 years or younger. In restrictive states, odds of firework injury were decreased (OR, 0.39; 95% CI, 0.24-0.63; *P* < .001) compared with unrestrictive states. In July, odds of firework injury were increased (OR, 17.94; 95% CI, 15.01-21.44; *P* < .001) vs other months.

**Figure.**
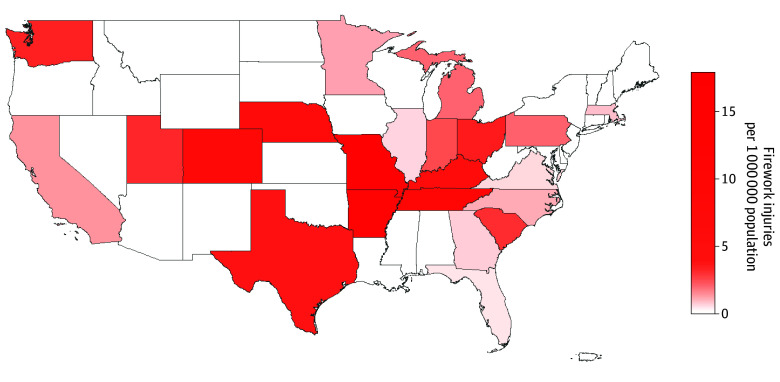
Firework Injury Prevalence by State Data are from the Pediatric Health Information System, 2015 to 2024. White regions indicate states not included in this study.

**Table.** Multilevel Logistic Regression Model

Characteristic	OR (SE) [95% CI]	*P* value
Intercept	0.01 (0.002) [0.00-0.01]	<.001
Patient age, per 1-y increase	1.09 (0.008) [1.07-1.11]	<.001
Patient gender		
Female	0.51 (0.049) [0.42-0.61]	<.001
Male	1 [Reference]	NA
Patient race^a^		
African American	1.37 (0.145) [1.12-1.69]	.003
White	1 [Reference]	NA
Other^b^	1.06 (0.133) [0.83-1.35]	.66
Unspecified	0.92 (0.225) [0.57-1.48]	.72
Patient residential area		
Rural	0.75 (0.125) [0.54-1.04]	.09
Urban	1 [Reference]	NA
Unknown	0.77 (0.294) [0.36-1.62]	.49
Legal state firework purchase age, y^c^		
≤15	1 [Reference]	NA
16	1.14 (0.451) [0.53-2.48]	.73
≥17	0.45 (0.169) [0.22-0.94]	.03
Illegal or partially legal	1.32 (0.744) [0.44-3.98]	.62
State firework consumer-purchase restrictions^d^		
Restrictive	0.39 (0.096) [0.24-0.63]	<.001
Unrestrictive	1 [Reference]	NA
Family income <state median		
No	1 [Reference]	NA
Yes	1.28 (0.114) [1.07-1.52]	.006
Injury occurring in July		
No	1 [Reference]	NA
Yes	17.94 (1.629) [15.01-21.44]	<.001

Abbreviations: NA, not applicable; OR, odds ratio.

^a^ Race was collected in the database as American Indian, Asian, Black, Pacific Islander, White, and other. Because the sample had few patients who were Asian, American Indian, Pacific Islander, and other, these categories were combined into 1 *other* category. Ethnicity was collected in the database as Hispanic or Latino or not Hispanic or Latino. Race and ethnicity were included as confounding factors.

^b^ Other race includes American Indian, Asian, Pacific Islander, and other.

^c^ Categories are based on data distribution.

^d^ Restrictive states were those that allowed consumers to purchase only nonprojectile and nonexplosive fireworks; unrestrictive states were those that allowed consumers to purchase all firework types or prohibited only sky lanterns.

## Discussion

In this cohort study, unrestrictive states that allowed consumer purchase of aerial and explosive fireworks experienced more injuries than states that restricted firework types. These findings align with previous research showing that injury rates increased after legislative relaxation of firework sales.^3,6^

Our study highlights important demographic disparities in firework-related injuries. Males, African American youths, and patients from lower-income households had significantly higher odds of firework-related injuries. Our results reinforce the documented seasonal spike in firework-related injuries in July, suggesting that prevention strategies should be intensified prior to Independence Day.

Study limitations include that the dataset had a subset of pediatric hospitals, limiting generalizability. Administrative coding may introduce misclassification bias. There may have been unaccounted confounders. Because of data size limitations, we could not perform longitudinal analysis accounting for across-year trends or policy changes during the study period. Additionally, we could not assess enforcement with state laws.

The findings support stronger regulatory measures for consumer fireworks, particularly restrictions on the sale of aerial and explosive devices. Complementary public health strategies targeting high-risk demographic groups are essential, including community-based safety initiatives and improved enforcement of existing regulations.

## Notes

Supplement 1. **eTable.** Patient Cohort Selection

Supplement 2.
Data Sharing Statement
