# Supplementary material for: State Firework Legislation and Pediatric Hand Trauma
Source: JAMA Netw Open. 2026 Jan 16;9(1):e2554594. doi: 10.1001/jamanetworkopen.2025.54594 (PMC12811801; doi:10.1001/jamanetworkopen.2025.54594)
Supplement: Supplement 1. — eTable. Patient Cohort Selection [file jamanetwopen-e2554594-s001.pdf]

Supplemental Online Content

Catanzaro MF, Kotsis SV, Pan W, Wang L, Chung KC. State firework legislation and pediatric hand trauma. *JAMA Netw Open*. 2026;9(1):e2554594.
doi:10.1001/jamanetworkopen.2025.54594

eTable. Patient Cohort Selection

This supplemental material has been provided by the authors to give readers additional information about their work.

© 2026 Catanzaro MF et al. *JAMA Network Open*.

eTable1. Patient Cohort Selection

Patients with Hand Injury Owing to Fireworks = Table A + Table B

Patients with Hand Injury Not Owing to Fireworks = Table B

**Table A. Firework Injury:**	
**ICD code**	**ICD code description**
W39.XXXA (ICD-10)<br>E923.0 (ICD-9)	Injuries caused by discharge of a firework, initial encounter<br>Accident caused by fireworks

**Table B. Hand Injury (one or more codes):**	
**ICD code**	**ICD code description**
T23.0-T23.7 (ICD-10)<br>944 (ICD-9)	Burn/corrosion of wrist and hand<br>Burn of wrist(s) and hand(s)
S64 (ICD-10)<br>955 (ICD-9)	Injury of nerves at wrist and hand level<br>Injury to peripheral nerve(s) of shoulder girdle and upper limb
S65 (ICD-10)<br>903 (ICD-9)	Injury of blood vessels at wrist and hand level<br>Injury to blood vessels of upper extremity
S68 (ICD-10)<br>881-887 (ICD-9)	Traumatic amputation of wrist/hand/fingers<br>Open wound of elbow, forearm and wrist
S66.100-S66.109 (ICD-10)<br>959.3-959.5 (ICD-9)	Injury of muscle/fascia/tendon to the wrist/hand<br>Elbow, forearm, wrist, hand and/or finger injury
S66.120-S66.129 (ICD-10)<br>913-915 (ICD-9)	Laceration of muscle/fascia/tendon to the wrist/hand<br>Superficial injury of elbow, forearm, wrist, hand, and/or fingers

© 2026 Catanzaro MF et al. *JAMA Network Open*.
