# Supplementary material for: State Firework Legislation and Pediatric Hand Trauma
Source: JAMA Netw Open. 2026 Jan 16;9(1):e2554594. doi: 10.1001/jamanetworkopen.2025.54594 (PMC12811801; doi:10.1001/jamanetworkopen.2025.54594)
Supplement: Supplement 2. — Data Sharing Statement [file jamanetwopen-e2554594-s002.pdf]

Data Sharing Statement

Catanzaro. State Firework Legislation and Pediatric Hand Trauma. *JAMA Netw Open*. Published online January 16, 2026. doi:10.1001/jamanetworkopen.2025.54594

Data Data for this study were obtained from the Pediatric Health Information System (PHIS), an administrative database that contains inpatient, emergency department, ambulatory surgery, and observation encounter-level data from not-for-profit, tertiary care pediatric hospitals in the US. These hospitals are affiliated with the Children's Hospital Association (Lenexa, KS). Data quality and reliability are assured through a joint effort between the Children's Hospital Association and participating hospitals. For the purposes of external benchmarking, participating hospitals provide discharge/ encounter data, including demographics, diagnoses, and procedures. Nearly all of these hospitals also submit resource utilization data (eg, pharmaceuticals, imaging, and laboratory) into PHIS. Data are deidentified at the time of data submission, and data are subjected to a number of reliability and validity checks before being included in the database. For this study, data from 49 hospitals were included. Participating hospitals in PHIS are the largest and most advanced children's hospitals in the US and constitute the most demanding standards of pediatric care in the US.
Data available: No
